# EFFECTS OF A SPECIFIC NARRATIVE GROUP PRACTICE ON HEALTH OUTCOMES OF CHINESE OLDER ADULTS: AN RCT STUDY

**DOI:** 10.1093/geroni/igac059.2149

**Published:** 2022-12-20

**Authors:** Esther Chow, Sai-fu Fung

**Affiliations:** City University of Hong Kong, Kowloon, Hong Kong; City University of Hong Kong, Kowloon, Hong Kong

## Abstract

In view of the rising chronicity during older adulthood, a specific narrative group practice is developed to improve well-being. Narrative therapy (NT), views people as experts in their own lives, and have accrued their life wisdom through their lifespan. To test its effects, a randomized controlled trial design was used to assess and compare outcomes between the intervention and waitlist-controlled group. A total of 157 older adults were recruited, 82 of which was randomly assigned to 9 intervention groups to receive four 2-hour NT sessions, recording difficult life circumstances, through narrative conversations, which made them stronger. Quantitative assessment of well-being, and depression were conducted at baseline (T0), at the end of treatment (T1), and at two (T2) and 8 months after treatment (T3). By employing the SEM parallel-process latent growth curve model, the results from the 4 waves of longitudinal data suggesting that the NT intervention have positive impact the participants, in particular the improved well-being of the participants contributed to the reduction of depressive symptoms with standardised estimate -0.502, p = 0.007. The estimated model fulfilled the criteria of mediocre fit, with TLI = 0.943; CFI = 0.969; RMSEA = 0.053. No adverse reaction was recorded in any of the cases mentioned at all study-sites. These findings have significant theoretical and practice implications for both health and social care professions. NT offers an effective practice to develop a strength- and meaning-based group practice and ground a new theory for to develop a constructive perspective to late life development.

